# Tuberculous meningoencephalitis concurrent with autoimmune glial fibrillary acidic protein astrocytopathy: a case report

**DOI:** 10.3389/fimmu.2026.1827602

**Published:** 2026-07-20

**Authors:** Yuan Deng, Zeyan Bao, SiDian Zhuang, Ting Wu, ShuiSheng Zhong

**Affiliations:** 1 Department of Neurology, Guangdong Sanjiu Brain Hospital, Guangzhou, China; 2 Department of Ultrasound Medicine, Guangdong Sanjiu Brain Hospital, Guangzhou, China

**Keywords:** case report, glial fibrillary acidic protein, overlap syndrome, paradoxical reaction, tuberculous meningoencephalitis

## Abstract

**Background:**

Tuberculous meningoencephalitis and autoimmune glial fibrillary acidic protein (GFAP) astrocytopathy display similar clinical features, cerebrospinal fluid (CSF) profiles, and neuroimaging patterns, which contribute to a high rate of misdiagnosis. Co-existence of these two diseases is extremely rare, and the clinical characteristics of this overlap syndrome remain poorly understood.

**Case presentation:**

A 67-year-old male patient was admitted with symptoms of fever, cognitive slowing, and limb weakness. Brain magnetic resonance imaging (MRI) showed multiple parenchymal signal abnormalities and leptomeningeal enhancement, associated with markedly increased intracranial pressure. The CSF showed pleocytosis, increased protein, decreased glucose, and decreased chloride levels. Simultaneously, a positive result was obtained from GeneXpert MTB/RIF, which is a diagnostic tool for tuberculous meningoencephalitis. In addition, GFAP antibodies were positive in both serum and CSF. Thus, a diagnosis of tuberculous meningoencephalitis and GFAP astrocytopathy was made. The patient showed significant clinical improvement after receiving antitubercular drugs and corticosteroids. Repeat MRI after steroid discontinuation demonstrated an increase in abnormal enhancing lesions. The lesions improved after glucocorticoid reintroduction.

**Conclusion:**

Tuberculous meningoencephalitis and GFAP astrocytopathy represent a rare overlap syndrome, and these cases are potentially susceptible to therapeutic paradoxical reactions, which are visible on imaging studies. Therapy for these cases involves concomitant anti-infective and immunomodulatory therapy, with consideration for the timing of steroid tapering and discontinuation.

## 1 Introduction

Tuberculous meningoencephalitis is a nonpurulent inflammatory disease caused by the invasion of *Mycobacterium tuberculosis* (MTB) into the meninges and brain tissue. The presenting symptoms of tuberculous meningoencephalitis include fever, headache, altered sensorium, nausea, vomiting, and seizures. Hyponatremia, hydrocephalus, hypoxic encephalopathy, and cerebral infarction are common complications. Delay in treatment is associated with a high mortality rate and a significant risk of disability (1, 2). Autoimmune glial fibrillary acidic protein (GFAP) astrocytopathy is a recently recognized form of autoimmune pathology of the central nervous system (CNS), involving the brain, meninges, spinal cord, and optic nerves. The diagnostic hallmark of this condition is the identification of anti-GFAP antibodies in the cerebrospinal fluid (CSF) and/or serum. Clinical manifestations include fever, headache, visual disturbances, neuropsychiatric symptoms, ataxia, motor disturbances, and autonomic disturbances. Radiologically, a distinctive feature of this condition is the linear radial enhancement perpendicular to the lateral ventricles. The nonspecific nature of the clinical presentation makes it difficult to diagnose this condition accurately. In most cases, it is confused with CNS infections, especially tuberculous meningitis (3–5). Notably, CNS infections and GFAP astrocytopathy are not entirely independent entities. Approximately 30-40% of patients with GFAP astrocytopathy report prodromal infectious symptoms before the onset of CNS symptoms. The most common symptoms include rhinorrhea, sore throat, and cough, while others develop the disease after a documented herpes virus infection (6, 7). However, cases of concurrent tuberculous meningoencephalitis and GFAP astrocytopathy remain rare. This study describes a case of a 67-year-old male diagnosed with both conditions of tuberculous meningoencephalitis and GFAP astrocytopathy. Following anti-tuberculosis and steroid therapy, his clinical symptoms improved, but radiological findings showed worsening. After adjustment of the steroid treatment, follow-up imaging demonstrated improvement.

## 2 Case presentation

A 67-year-old male was admitted on July 10, 2025, with a 2-week history of recurrent fever and a one-week history of mental dullness and limb weakness. Two weeks before admission, the patient developed an unexplained fever with a peak temperature of 39.3 °C. Treatment with intravenous ceftriaxone at a local hospital produced no clinical improvement. One week before admission, he developed mental dullness, apathy, memory impairment, and generalized limb weakness, eventually becoming unable to walk. The patient’s past medical history was noncontributory. On examination, the patient appeared apathetic and responded slowly. His recent memory and calculation abilities were impaired, whereas orientation and judgment remained intact. Muscle strength was graded 3 out of 5 in all four limbs. Neck stiffness was present, with a chin to chest distance of four fingerbreadths. Both Kernig sign and Brudzinski sign were negative.

On the second hospital day, a lumbar puncture was performed. Despite prior osmotic dehydration with mannitol, the opening pressure remained elevated (250 mmH_2_O). CSF analysis showed markedly elevated protein levels (2.19 g/L, reference 0.15–0.45 g/L) and nucleated cell counts (310 × 10^6^/L, reference ≤ 5 × 10^6^/L), with lymphocytic predominance, along with reduced glucose (2.2 mmol/L, reference 2.5–4.4 mmol/L) and chloride (96.4 mmol/L, reference 120–130 mmol/L) (**Table 1**). Tuberculosis-related testing demonstrated a borderline serum T-SPOT.TB result and a positive CSF GeneXpert MTB/RIF assay. However, serum anti-tuberculosis antibodies, CSF acid-fast staining, and CSF metagenomic next-generation sequencing (mNGS) were all negative. Further, autoimmune antibody testing using a cell-based indirect immunofluorescence assay demonstrated positive GFAP antibodies in both serum (1:100) and CSF (1:10) (**Figure 1**), whereas antibodies to aquaporin-4 (AQP4), myelin oligodendrocyte glycoprotein (MOG), and myelin basic protein (MBP) were negative. Tumor screening revealed a mildly elevated total prostate-specific antigen (6.11 ng/mL, reference ≤ 4 ng/mL), while alpha-fetoprotein, carcinoembryonic antigen, human chorionic gonadotropin, squamous cell carcinoma antigen, and carbohydrate antigen 19–9 were within normal limits. Serum electrolyte analysis revealed hyponatremia, with a serum sodium level of 129.4 mmol/L. Complete blood count, procalcitonin, blood cultures, and fungal cultures were unremarkable. Brain magnetic resonance imaging (MRI) demonstrated multiple abnormal cortical and subcortical signal changes involving the right frontal insular and occipital lobes, abnormal sulcal signals in the right cerebral hemisphere, and multiple linear and punctate leptomeningeal enhancements on contrast-enhanced imaging (**Figure 2**). Chest computed tomography showed partial consolidation in both lower lungs and subpleural fibrotic lesions at both lung apices.

**Table 1:** Laboratory results.

Contents	July 17, 2025	October 21, 2025
Opening pressure (mmH2O)	250	100
CSF WBC (10^6/L)	310	130
CSF Protein (g/L)	2.19	1.56
CSF Glucose (mmol/L)	2.2	3.3
CSF Chlorine (mmol/L)	96.4	116.5
CSF Modified acid-fast staining	(-)	(-)
CSF GeneXpert MTB/RIF	(+)	(-)
CSF GFAP-ab	1:10	(-)
Serum GFAP -ab	1:10	(-)

**Figure 1:**
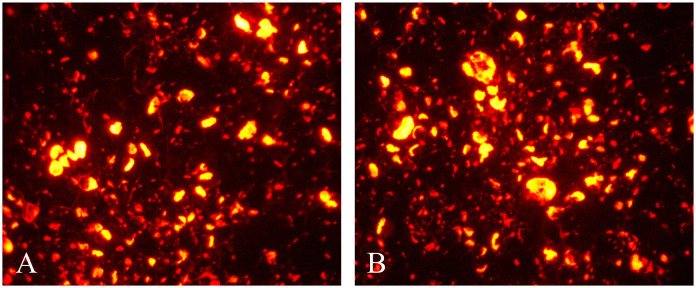
Cell−based assay for GFAP antibodies. Positive staining in **(A)** serum (1:100) and **(B)** CSF (1:10).

**Figure 2:**
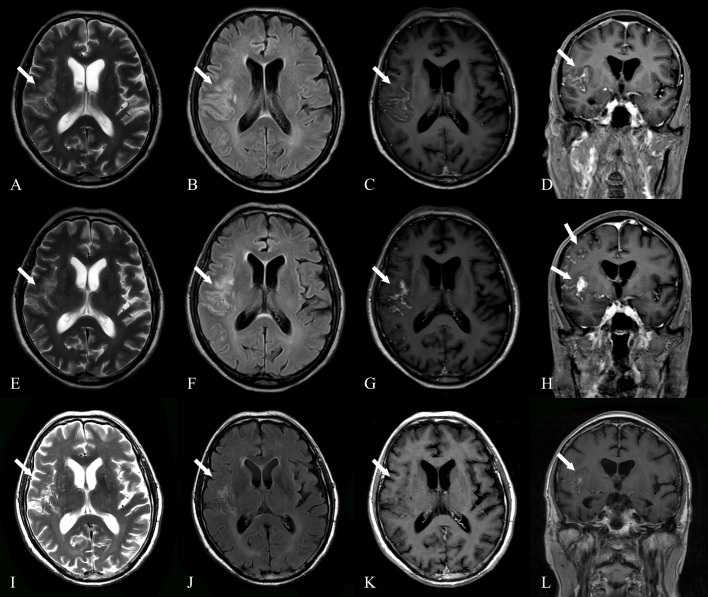
Brain MRI at different time points. Initial MRI showed multiple abnormal hyperintense lesions on T2-weighted **(A)** and FLAIR **(B)** images involving the right frontal, insular, and occipital cortex and subcortical white matter, together with abnormal sulcal hyperintensity in the right cerebral hemisphere. Axial post-contrast T1-weighted **(C)** and coronal post-contrast T1-weighted **(D)** images demonstrated multiple linear and punctate leptomeningeal enhancements. At the 1-month follow-up, T2-weighted **(E)** and FLAIR **(F)** images showed mildly increased hyperintense lesions compared with baseline. Axial post-contrast T1-weighted **(G)** and coronal post-contrast T1-weighted **(H)** images demonstrated newly developed nodular enhancement and more prominent leptomeningeal enhancement. At the 3-month follow-up, T2-weighted **(I)** and FLAIR **(J)** images demonstrated partial resolution of the previously observed lesions. Axial post-contrast T1-weighted **(K)** and coronal post-contrast T1-weighted **(L)** images showed marked regression of the previously enhanced lesions.

Consequently, the patient was diagnosed with tuberculous meningoencephalitis concurrent with GFAP astrocytopathy. Antituberculous therapy was initiated consisting of isoniazid (0.6 g/day), rifampicin (0.45 g/day), ethambutol (0.75 g/day), pyrazinamide (1.5 g/day), and moxifloxacin (0.4 g/day), together with intravenous methylprednisolone (250 mg/day for 5 days, followed by 125 mg/day for 3 days), which was subsequently transitioned to oral prednisone (60 mg/day). The patient showed signs of clinical improvement after ten days of treatment, including improved responsiveness to stimuli, appropriate verbal communication, normalization of body temperature, absence of meningeal signs, and increased limb strength enabling assisted walking. After one month, the strength in the muscles was fully restored, and the patient was able to walk on his own; however, the patient still suffered from impairments in memory and calculation. The Mini-Mental State Examination (MMSE) score was 23, and the Montreal Cognitive Assessment (MoCA) score was 18. Prednisone was discontinued at that time. Repeat brain MRI showed mild progression of abnormal signal intensity at the original lesion sites, with newly developed nodular enhancement and more prominent leptomeningeal enhancement. Consequently, oral prednisone (60 mg/day) was reintroduced alongside ongoing antituberculous therapy, maintained for 4 weeks, and then gradually tapered by 10 mg every 2 weeks. At the 3-month follow-up, memory and calculation abilities showed no significant improvement, whereas fever and limb weakness did not recur. Repeat lumbar puncture demonstrated decreased opening pressure (100 mmH_2_O), reduced nucleated cell count (130 × 10^6^/L), and lower protein levels (1.56 g/L), with increased glucose (3.3 mmol/L) and chloride (116.5 mmol/L) compared with prior measurements (**Table 1**). GFAP antibodies in both serum and CSF had become negative. Follow-up brain MRI showed marked improvement of the previously enhanced lesions (**Figure 2**). Oral prednisone tapering was continued during follow-up.

## 3 Discussion

Tuberculous meningoencephalitis is among the most severe forms of extrapulmonary tuberculosis. Without timely treatment, it may lead to impaired consciousness, focal neurological deficits, or death (1). Given the low positivity rate of direct CSF pathogen detection, diagnosis often depends on comprehensive clinical evaluation. According to the 2010 international consensus diagnostic criteria for tuberculous meningitis (8), this patient fulfilled the diagnostic requirements, with evidence of meningitis, including fever, altered mental status, and meningeal signs, together with a positive CSF GeneXpert MTB/RIF result. In addition, the patient’s serum T−SPOT.TB result was borderline positive. A recent large retrospective cohort study demonstrated that both borderline (5–7 spots) and positive (≥8 spots) T−SPOT.TB results are associated with an increased risk of prevalent and incident tuberculosis disease (9), further supporting the possibility of tuberculosis infection in this case. Although mNGS did not detect MTB nucleic acids, this result may reflect a low mycobacterial burden in CSF. A meta-analysis reported that while mNGS has high specificity (99%) for tuberculous meningitis diagnosis, its sensitivity remains limited (62%) (10).

Importantly, the diagnosis of tuberculous meningoencephalitis should be made cautiously when direct microbiological evidence is absent, as GFAP astrocytopathy can closely mimic tuberculous meningoencephalitis with respect to CSF inflammatory profiles, neuroimaging findings, and clinical presentation. In particular, when characteristic radial perivascular enhancement or confirmatory antibody testing is lacking, GFAP astrocytopathy may be misdiagnosed as tuberculous meningitis, with reported misdiagnosis rates ranging from 4.5% to 35.7% (11).

This patient also presented with hyponatremia (serum sodium 129.4 mmol/L). Hyponatremia is common in tuberculous meningitis and is usually associated with syndrome of inappropriate antidiuretic hormone secretion or cerebral salt wasting syndrome. A recent meta-analysis reported a pooled hyponatremia prevalence of 52% in tuberculous meningitis, with a pooled mean serum sodium level of 131.08 mEq/L (12). Hyponatremia has likewise been reported frequently in autoimmune GFAP astrocytopathy, occurring in 57% of patients during hospitalization in one retrospective study (13). Taken together, these findings indicate that hyponatremia may represent a shared clinical feature of both tuberculous meningoencephalitis and GFAP astrocytopathy, although it has limited specificity for distinguishing between the two conditions.

Given these overlapping clinical features, GFAP astrocytopathy was included early in the differential diagnosis for this patient, and GFAP antibody testing was performed in both serum and CSF. CSF and serum samples were both positive for GFAP antibodies, with a CSF titer of 1:10. In a 2025 study of Chinese patients, Wu et al. reported a median CSF GFAP antibody titer of 1:10 (range 1:1–1:320) (14), similar to the value observed in our patient. Corticosteroid responsiveness is a well-recognized feature of GFAP astrocytopathy (4). In this patient, despite continued antituberculous therapy, radiological worsening occurred after glucocorticoid withdrawal and improvement followed reintroduction. Furthermore, both CSF and serum GFAP antibodies converted to negative during follow-up. These convergent findings support the diagnosis of a rare overlap syndrome involving tuberculous meningoencephalitis and GFAP astrocytopathy.

GFAP is an intermediate filament protein expressed in astrocytes that functions not only as an astrocytic marker but also as a structural element essential for maintaining cellular integrity, blood-brain barrier stability, and synaptic regulation (15). Current evidence indicates that the immunopathogenesis of GFAP astrocytopathy is driven primarily by cytotoxic T-cell-mediated immune responses rather than by a direct pathogenic effect of GFAP antibodies. Brain biopsy studies have shown prominent perivascular inflammatory cell infiltration and microglial activation in patients with GFAP astrocytopathy (16), while CD8^+^ T-cell infiltration adjacent to degenerating neurons and astrocytes has also been documented (17). In addition, inflammatory pathways may influence disease severity, as increased levels of the NLRP3 inflammasome and proinflammatory cytokines (IL-1β, IL-6, and IL-17) in the CSF have been shown to correlate with clinical severity in GFAP astrocytopathy (18). It has also been proposed that astrocytic injury followed by exposure of intracellular antigens after infection may promote aberrant autoimmune activation (19). In support of this hypothesis, GFAP has been associated with both viral and bacterial infections, including human herpesvirus 7 encephalitis and Brucella infection (6, 20).

Although there is no direct evidence that tuberculous meningitis can cause GFAP astrocytopathy, their coexistence in this case raises the possibility that both tuberculous meningoencephalitis and GFAP astrocytopathy could share an immunopathological mechanism. Tuberculous meningoencephalitis could be considered a triggering cause for GFAP astrocytopathy through mechanisms of molecular mimicry or post-infectious immune activation. For example, Mycobacterium tuberculosis infection has been linked to an inflammatory response that affects both astrocytes and the blood–brain barrier, thus exposing GFAP epitopes. The pro-inflammatory mediators induced in tuberculosis infection, including cytokines, matrix metalloproteinases, and adhesion molecules, have been shown to play a role in the recruitment of immune cells in the CNS and enhance inflammatory signaling in the CNS (21). Additionally, delayed-type hypersensitivity to bacterial antigens can enhance T cell-mediated immunity and subsequent cytokine production (22). It is worth noting that these mechanisms are still speculative in this context, and we cannot exclude the possibility that tuberculous meningoencephalitis and GFAP astrocytopathy coexist coincidentally.

The patient initially improved following combined antituberculous therapy and glucocorticoid treatment. However, during follow-up, radiological worsening was observed despite ongoing antituberculous therapy. This deterioration raised concern for either a paradoxical reaction associated with tuberculous meningoencephalitis or relapse of GFAP astrocytopathy. Paradoxical reactions in tuberculous meningoencephalitis are defined as deterioration in the clinical or radiological condition despite appropriate antituberculous treatment (23). They affect approximately one-third of patients and usually occur within the first three months of treatment (24). The prevailing hypothesis attributes this phenomenon to an exaggerated host inflammatory response to mycobacterial antigens released during effective antimicrobial therapy (23). Management of paradoxical reactions generally involves continuation of antituberculous therapy together with appropriate immunomodulatory treatment, particularly glucocorticoids (25). In our case, the favorable radiological response to glucocorticoid reintroduction suggests that autoimmune inflammatory mechanisms may have played an important role in the disease course.

At the same time, GFAP astrocytopathy is also known to relapse during steroid tapering or withdrawal. Previous studies have reported relapse rates ranging from 20% to 50%. Relapse may occur during glucocorticoid tapering or even despite ongoing treatment (4, 26). In this case, radiological deterioration after steroid withdrawal further suggested that the autoimmune inflammatory response had not been completely suppressed. Therefore, in patients with coexisting tuberculous meningoencephalitis and GFAP astrocytopathy, distinguishing paradoxical reactions from autoimmune relapse may be challenging.

Wei et al. reported a case of GFAP astrocytopathy with overlapping autoimmune syndrome successfully treated with intravenous methylprednisolone followed by mycophenolate mofetil maintenance therapy (27). In contrast, our patient had active tuberculous meningoencephalitis, making escalation of immunosuppressive therapy particularly challenging, as excessive immunosuppression may aggravate the underlying infection. Similar concerns regarding the risk of infection during long-term immunosuppression have been reported in other immune-mediated diseases, including a recent case of tuberculous meningitis developing during prolonged immunosuppression (28). In this patient, only short-term glucocorticoids were administered, and the radiological worsening after withdrawal with subsequent improvement upon reintroduction suggests that more gradual glucocorticoid tapering may be beneficial in such overlap cases.

## 4 Conclusion

In conclusion, we describe a rare case of tuberculous meningoencephalitis complicated by autoimmune GFAP astrocytopathy. The present case reminds all clinicians treating tuberculous meningitis to include GFAP astrocytopathy in the list of possible differential diagnoses and to be aware of the possibility of co-occurrence of both conditions. Such cases might also be associated with an increased risk of paradoxical worsening of radiological abnormalities during treatment, which may require a therapeutic regimen combining anti-tuberculosis therapy and immunomodulation. Any attempt to taper immunotherapy, including glucocorticoids, should be made cautiously to avoid any possibility of relapse or deterioration of the patient’s health from reduced immunosuppression.

## Data Availability

All data generated or analyzed during this study are included in this published article and its supplementary information files.

## References

[1] Wilkinson RJ Rohlwink U Misra UK van Crevel R Mai NTH Dooley KE . Tuberculous meningitis. Nat Rev Neurol. (2017) 13:581–98. doi: 10.1038/nrneurol.2017.120 28884751

[2] Schaller MA Wicke F Foerch C Weidauer S . Central nervous system tuberculosis. Clin Neuroradiol. (2019) 29:3–18. doi: 10.1007/s00062-018-0726-9 30225516

[3] Zhao W Gong L Wang J Chen Z Li Z Su Q . Case reports: three cases of autoimmune glial fibrillary acidic protein astrocytosis in disguise. Front Immunol. (2025) 16:1546372. doi: 10.3389/fimmu.2025.1546372 40735311 PMC12303806

[4] Kunchok A Zekeridou A McKeon A . Autoimmune glial fibrillary acidic protein astrocytopathy. Curr Opin Neurol. (2019) 32:452–8. doi: 10.1097/WCO.0000000000000676 30724768 PMC6522205

[5] Bai R An L Du W Wang Z Qi X Liu J . Autoimmune glial fibrillary acidic protein astrocytopathy misdiagnosed as intracranial infectious diseases: case reports and literature review. Front Immunol. (2025) 16:1519700. doi: 10.3389/fimmu.2025.1519700 39911384 PMC11794125

[6] Li YX Wang RY . Autoimmune glial fibrillary acidic protein astrocytopathy following human herpesvirus-7 infection: a case report. Brain Inj. (2026) 40:207–10. doi: 10.1080/02699052.2025.2596225 41314968

[7] Cheng P Huang W Yang M Chen Z Geng Y Zhang X . Autoimmune GFAP astrocytopathy after viral encephalitis: a case report of bimodal overlapping encephalitis. Front Immunol. (2023) 14:1258048. doi: 10.3389/fimmu.2023.1258048 37781407 PMC10535097

[8] Marais S Thwaites G Schoeman JF Török ME Misra UK Prasad K . Tuberculous meningitis: a uniform case definition for use in clinical research. Lancet Infect Dis. (2010) 10:803–12. doi: 10.1016/S1473-3099(10)70138-9 20822958

[9] Skouvig Pedersen O Sperling S Koch A Lillebaek T Dahl VN Fløe A . Evaluating stratified T-SPOT.TB results for diagnostic accuracy in tuberculosis disease: a retrospective cohort study with sensitivities, specificities, and predictive values. Clin Microbiol Infect. (2025) 31:808–17. doi: 10.1016/j.cmi.2025.01.002 39793964

[10] Xiang ZB Leng EL Cao WF Liu SM Zhou YL Luo CQ . A systematic review and meta-analysis of the diagnostic accuracy of metagenomic next-generation sequencing for diagnosing tuberculous meningitis. Front Immunol. (2023) 14:1223675. doi: 10.3389/fimmu.2023.1223675 37822937 PMC10562686

[11] Liang Y Wang G Li B Li G Zeng H . Autoimmune glial fibrillary acidic protein astrocytosis mimicking tuberculous meningitis: a retrospective study. J Neurol. (2023) 270:4860–7. doi: 10.1007/s00415-023-11818-8 37338614 PMC10511357

[12] Hieu TH Hashan MR Morsy S Tawfik GM Cucè F Sharma A . Hyponatremia in tuberculous meningitis: a systematic review and meta-analysis. Indian J Tuberc. (2021) 68:516–26. doi: 10.1016/j.ijtb.2021.06.004 34752323

[13] Kimura A Takekoshi A Yoshikura N Hayashi Y Shimohata T . Clinical characteristics of autoimmune GFAP astrocytopathy. J Neuroimmunol. (2019) 332:91–8. doi: 10.1016/j.jneuroim.2019.04.004 30991306

[14] Wu T Zhang H Gao C Yu Q Fan M Zhang L . Glial fibrillary acidic protein astrocytopathy based on a two-center Chinese cohort study. Ann Clin Transl Neurol. (2025) 12:1813–22. doi: 10.1002/acn3.70118 40641094 PMC12455873

[15] McKeon A Benarroch EE . Glial fibrillary acid protein: functions and involvement in disease. Neurology. (2018) 90:925–30. doi: 10.1212/WNL.0000000000005534 29653988

[16] Long Y Liang J Xu H Huang Q Yang J Gao C . Autoimmune glial fibrillary acidic protein astrocytopathy in Chinese patients: a retrospective study. Eur J Neurol. (2018) 25:477–83. doi: 10.1111/ene.13531 29193473

[17] Yuan Z Li H Huang L Fu C Chen Y Zhi C . CD8+ T‐cell predominance in autoimmune glial fibrillary acidic protein astrocytopathy. Eur J Neurol. (2021) 28:2121–5. doi: 10.1111/ene.14778 33590610

[18] Luo Y Yan W Zhou Z Liu B Wang Z Chen J . Elevated levels of NLRP3 in cerebrospinal fluid of patients with autoimmune GFAP astrocytopathy. Front Neurol. (2019) 10:1019. doi: 10.3389/fneur.2019.01019 31681133 PMC6812676

[19] Liao Y Wen L Zheng R Shen Y Ha TA Lin M . Novel perspectives focused on the relationship between Herpesvirus Encephalitis and Anti-GFAP-Antibody-Positive Astrocytopathy. Mol Neurobiol. (2025) 62:6179–94. doi: 10.1007/s12035-024-04660-0 39731639

[20] He Q Liu J Zhu Z Tang Y Long L Hu K . Case report: revealing a special and rare autoimmune GFAP astrocytopathy in the spinal cord succeeding neurobrucellosis infection. Front Immunol. (2022) 13:950522. doi: 10.3389/fimmu.2022.950522 35990675 PMC9389076

[21] Barnacle JR Davis AG Wilkinson RJ . Recent advances in understanding the human host immune response in tuberculous meningitis. Front Immunol. (2024) 14:1326651. doi: 10.3389/fimmu.2023.1326651 38264653 PMC10803428

[22] Chen X Wang Q Li J Yang H Chen S . Concurrent tuberculous meningoencephalitis and anti-NMDAR encephalitis: a case report. Front Neurol. (2022) 13:870607. doi: 10.3389/fneur.2022.870607 35873772 PMC9301103

[23] Bell LCK Breen R Miller RF Noursadeghi M Lipman M . Paradoxical reactions and immune reconstitution inflammatory syndrome in tuberculosis. Int J Infect Dis. (2015) 32:39–45. doi: 10.1016/j.ijid.2014.12.030 25809754

[24] Singh AK Malhotra HS Garg RK Jain A Kumar N Kohli N . Paradoxical reaction in tuberculous meningitis: presentation, predictors and impact on prognosis. BMC Infect Dis. (2016) 16:306. doi: 10.1186/s12879-016-1625-9 27329253 PMC4915108

[25] Garg RK Malhotra HS Kumar N . Paradoxical reaction in HIV negative tuberculous meningitis. J Neurol Sci. (2014) 340:26–36. doi: 10.1016/j.jns.2014.03.025 24680563

[26] Xiao J Chen X Shang K Tang Y Chen M Deng G . Clinical, neuroradiological, diagnostic and prognostic profile of autoimmune glial fibrillary acidic protein astrocytopathy: a pooled analysis of 324 cases from published data and a single-center retrospective study. J Neuroimmunol. (2021) 360:577718. doi: 10.1016/j.jneuroim.2021.577718 34600199

[27] Wei W Chen M Meng L . Case report: autoimmune glial fibrillary acidic protein astrocytopathy with overlapping autoimmune syndrome. Front Immunol. (2024) 15:1485374. doi: 10.3389/fimmu.2024.1485374 39464885 PMC11512446

[28] Panda S Narayanan Kaladi S Jagzape TB . Immunosuppressive therapy in Takayasu arteritis: a tightrope walk between inflammation and infection. Cureus. (2025) 17:e97954. doi: 10.7759/cureus.97954 41458842 PMC12743592

